# Efficacy and safety of postural intervention on prevention of deep venous thrombosis of lower extremity in postpartum women with pregnancy-induced hypertension: A protocol for systematic review and meta-analysis

**DOI:** 10.1097/MD.0000000000024959

**Published:** 2021-03-26

**Authors:** Jie Chen, Lijuan Guo, Shuangxi Li, Yuqin Shi

**Affiliations:** a Department of Interventional Medicine, The First Hospital of Lanzhou University; b Intensive Care Unit, Gansu Provincial Hospital; c Department of International Medical Services, Gansu Provincial Maternity and Child-Care Hospital, Lanzhou, Gansu Province, China.

**Keywords:** deep venous thrombosis, meta-analysis, postural intervention, pregnancy-induced hypertension, protocol, systematic review

## Abstract

**Background:**

Deep venous thrombosis (DVT) is a relatively serious complication commonly seen in pregnant women, especially parturients with pregnancy-induced hypertension (PIH), whose incidence of DVT is higher. Because it can lead to pulmonary embolism (PE) and endanger the lives of patients, it has been paid much attention in clinic. Nursing plays an important role in the prevention and treatment of DVT. Early posture intervention can prevent postpartum DVT in hypertensive pregnant women, which has certain clinical value, but there is no evidence of evidence-based medicine. This study aims to systematically study the effectiveness and safety of postural intervention on prevention of deep venous thrombosis of lower extremity in postpartum women with PIH.

**Methods:**

Use the computer to retrieve the English databases (PubMed, Embase, Web of Science, the Cochrane Library) and the Chinese databases (China Knowledge Network, Wanfang, Weipu, China Biomedical Database), in addition to the manual retrieval of Baidu academic, Google academic, from the construction of database to December 2020, for randomized controlled clinical studies of postural intervention on prevention of deep venous thrombosis of lower extremity in postpartum women with PIH. Two researchers independently extracted the data and evaluated the quality of the included research, and used RevMan5.3 software to do meta-analyze of the included literature.

**Results:**

This study assessed the efficacy and safety of potential intervention on prevention of deep venous thrombosis of lower extremities with lower extremity hypertension by mean velocity of femoral venous flow in the lower extremities, lower extremity skin status, swelling level, and pain condition, lower extremity deep venous thrombosis rate, and incidence of pulmonary embolism.

**Conclusion:**

This study will provide reliable evidence-based evidence for the clinical application of postural intervention on prevention of deep venous of lower extremity in Postpartum women with PIH.

**OSF Registration number:**

DOI 10.17605/OSF.IO/4NPKW.

## 1 Introduction

Venous thromboembolism (VTE) is widely recognized as important complications of pregnancy. Indeed, VTE remains one of the leading causes of maternal mortality in developed countries.^[1–2]^ Pregnant and postpartum patients have a fourfold increased risk of VTE compared to the general population, and VTE accounts for 9% of maternal deaths in the United States.^[3–5]^ VTE includes deep venous thrombosis (DVT) and pulmonary embolism (PE), and DVT is becoming more and more common in clinical practice, which attracts much attention because of its tendency to endanger the life safety of patients with PE. Parturients with pregnancy-induced hypertension (PIH) have a higher chance of developing lower extremity DVT due to their abnormally increased blood pressure, increased resistance to blood flow, and increased blood viscosity, combined with the postpartum maternal need for bed rest and decreased limb movement,^[6]^ which are detrimental to postpartum recovery. Nursing plays an important role in the prevention and treatment of DVT,^[7–8]^ among them, early posture intervention is of great significance to prevent lower limb DVT in pregnant women with PIH.

At present, there are several randomized controlled findings^[9–11]^ that suggest that postural intervention on prevention of deep venous thrombosis of lower extremity in postpartum women with PIH can effectively prevent the occurrence of postpartum lower extremity swelling and deep venous thrombosis in pregnant women with PIH, which is helpful for promoting the postpartum recovery of pregnant women, has important practical significance, and deserves to be promoted in the clinical range. However, various clinical trials have differences in the research protocols and efficacy, and so on, leading to mixed findings, which to some extent affects the promotion of this therapy. Therefore, this research is to systematically evaluate the effectiveness and safety of postural intervention on prevention of deep venous of lower extremity in postpartum women with PIH. This paper provides reliable reference for postural intervention on prevention of deep venous of lower extremity in postpartum women with PIH.

## 2 Methods

### 2.1 Protocol register

This protocol of systematic review and meta-analysis has been drafted under the guidance of the preferred reporting items for systematic reviews and meta-analyses protocols.^[12]^ Moreover, it has been registered on open science framework (OSF) (Registration number: DOI 10.17605/OSF.IO/4NPKW).

### 2.2 Ethics

We have no patient recruitment and collection of personal information for this protocol, so the approval of the ethics committee is not required.

### 2.3 Eligibility criteria

#### 2.3.1 Types of studies

We collected all available randomized controlled trails (RCTs) on postural intervention on prevention of deep venous thrombosis of lower extremity in postpartum women with PIH. Whether or not blind method was used; language was limited to Chinese and English; publication status was unlimited.

#### 2.3.2 Study subjects

1. Pregnant women diagnosed with PIH, meeting the diagnostic criteria of PIH in the Guidelines for the diagnosis and treatment of PIH (2015)^[13]^;
2. The blood pressure lowering medication regimen was consistent in the 2 groups;
3. Limb movement was not restricted;
4. The nationality, race, age, gestational week, delivery time, delivery mode, and so on of pregnant women are not limited.

#### 2.3.3 Interventions

The control group received routine care (including preaching, holter monitoring, oxygen inhalation therapy, hypertension control, diet care, activity care, etc.), and specific care modalities were not limited; the observation group, on the basis of the same routine care as the control group, plus early postural interventions (recumbent position with head low and feet high and with the head biased to 1 side, massage of the lower extremities, early passive, and active activity instruction, etc.).

#### 2.3.4 Outcome indicators

Lower extremity femoral venous flow mean velocity; lower extremity skin status, swelling level, pain condition; lower extremity deep venous thrombosis rate; and incidence of PE.

### 2.4 Exclusion criteria

1. Duplicate published papers;
2. Literature published as an abstract or with incomplete data, and articles for which complete data could not be obtained after contacting the authors;
3. Literature without relevant outcome indicators;
4. Literature in which patients with gestational diabetes mellitus and other pregnancy complications;
5. Literature in which patients with a previous history of DVT;

### 2.5 Search strategy

Taking “ ti wei gan yu” (postural intervention), “ren shen qi gao xue ya” “(gestational hypertension),” ren gao zheng “(PIH),”shen jing mai xue shuan” (deep venous thrombosis), and so on as Chinese search terms, the Chinese databases were searched, including China Knowledge Network, Wanfang data knowledge service platform, Weipu Information Chinese Journal Service platform (VIP), China Biomedical Database; Taking “postural intervention,” “deep venous thrombosis,” “deep vein thrombosis,” “pregnancy,” “pregnant,” “postpartum,” “hypertension,” and so on as English search terms, the English databases were searched, including PubMed, Embase, Web of Science, the Cochrane Library, in addition manual retrieval was conducted in Baidu academic, Google academic. Search time was from the establishment of the database to December 2020, for all the domestic and foreign literatures about postural intervention on prevention of deep venous thrombosis of lower extremity in postpartum women with PIH. For example, PubMed retrieval strategy is shown in Table 1.

**Table 1:** Search strategy in PubMed database.

Number	Search terms
#1	deep vein thrombosis [Title/Abstract]
#2	deep venous thrombosis [Title/Abstract]
#3	#1 OR #2
#4	postural intervention [Title/Abstract]
#5	pregnant [Title/Abstract]
#6	pregnancy [Title/Abstract]
#7	postpartum [Title/Abstract]
#8	#4 OR#5 OR #6 OR #7
#9	hypertension [Title/Abstract]
#10	#3 AND #8 AND #9

### 2.6 Data screening and extraction

Referring to the method of research selection in version 5.0 of the Cochrane collaboration Network system Evaluator Manual, according to the PRISMA flow chart, the 2 researchers used the EndNote X9 document management software to independently screen and check the literature according to the above inclusion and exclusion criteria, and check each other, if there were different opinions, negotiate with a third party to resolve the differences. At the same time, Excel 2013 was used to extract relevant information, including:

1. Clinical studies (title, first author, month of publication, sample size, mean age, mean gestational week, mode of delivery, mean number of births);
2. Interventions (medication, dosage, frequency, and course of hypertension in the control and observation groups; other usual care modalities in the control and observation groups; implementation plans of early postural intervention in the observation group);
3. Elements of risk bias assessment in RCTs;
4. Outcome indicators.

**Figure 1:**
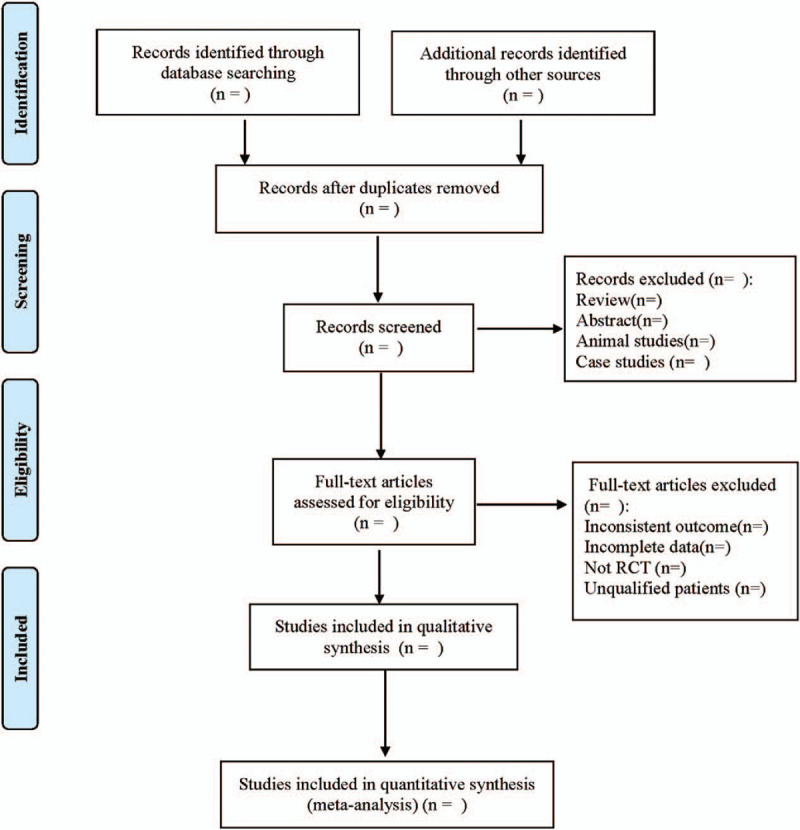
Flow diagram.

The literature screening process is shown in Figure 1.

### 2.7 Literature quality assessment

Use the Cochrane collaboration's tool for assessing risk of bias to do the risk of bias assessment of included studies. According to the performance of the included literatures in the above evaluation items, 2 researchers will give judgments like low risk, unclear, or high risk judgments 1 by 1, and cross-check after completion respectively. In case of any disagreement, discussion will be carried out. If no agreement can be reached between the 2, discussion will be made with the researchers in the third party.

### 2.8 Statistical analysis

#### 2.8.1 Data analysis and processing

The RevMan 5.3 software provided by the Cochrane Collaboration will be used for statistical analysis. We assumed that the results of different studies depend on the sample variables, covariables, and other factors studied. Fixed effects analysis allows only inferences similar to those contained in the meta-analysis, and random effects analysis allows a wider range of inferences. The overall rate was estimated to be a 95% confidence interval as well as the effect sizes on the meta-regression model. When this interval contained the value of 1, and the effect had a *P* value greater than 5%, we considered it to have no effect, *I*^*2*^ index was used to measure the heterogeneity of different study results. The index can vary between negative values (assuming 0%) to 100%. It is suggested that up to 25% is a small degree of heterogeneity, up to 50% is a medium degree, and 75% and above are high degree.

#### 2.8.2 Dealing with missing data

If data was missing from this article, please email the author for more information. If the author cannot be reached, or if the author has lost relevant data, descriptive analysis, rather than meta-analysis, will be performed.

#### 2.8.3 Subgroup analysis

Subgroup analyses were performed according to the mode of delivery, which can be divided into 2 types: normal delivery and cesarean section; subgroup analyses were performed according to the number of births, which can be divided into 2 types: primipara and pluripara; subgroup analyses were performed according to hypertension grade; subgroup analyses were performed according to gestational weeks.

#### 2.8.4 Sensitivity analysis

Because we noticed significant heterogeneity of the data, we performed a sensitivity analysis by stepwise exclusion of each study.

#### 2.8.5 Assessment of reporting biases

Publication bias was evaluated using the funnel plot and analyses were performed. Moreover, Egger and Begg test were used for the evaluation of potential publication bias.

#### 2.8.6 Evidence quality evaluation

We will choose to assess the quality of evidence by using the Grading of Recommendations Assessment, Development, and Evaluation, which contains 5 domains, including bias risk, consistency, directness, precision, and publication bias. We would rate the quality of evidence as high, moderate, low, and very low.

## 3 Discussion

Studies have shown that certain conditions have been associated with the high risk of pregnancy related DVT. These include inherited or acquired thrombophilias, a previous history of thrombosis, antiphospholipid syndrome, lupus, heart disease, sickle cell disease, etc.^[14]^ Other independent risk factors are age 35 and older, null parity, multiple gestations, obesity, and immobility, these increase the risk 1.5 to 2 fold.^[15–17]^ DVT of the lower extremities is mainly due to the abnormal clotting of blood in the deep venous lumen to form a thrombus, which leads to venous reflux disorders caused by venous vascular obstruction, and easily leads to sensory and motor nerve disorders in the lower extremities of patients, manifested by altered skin status of the lower extremities, swelling, pain, itching, etc., and in severe cases, it even triggers PE, threatening the life safety of patients. According to 3 major factors of lower extremity deep venous thrombosis proposed by Virchow, including slow venous flow velocity and stasis, vessel wall damage, and blood hypercoagulation,^[18–19]^ various coagulation factors, such as II, V, VII, VIII, etc., are significantly increased in the maternal blood during the third trimester, and the pregnant women with PIH have a high erythrocyte aggregation index and weak deformability, all of which can make their blood stagnant and increase the resistance to blood flow, and make their blood chronically hypercoagulable. And because of multiple options for cesarean section in the pregnant women with PIH, surgical trauma, or intravenous fluids can injure the venous vascular wall. And to enable a successful delivery of pregnant women with PIH, continuous epidural anesthesia is required to dilate the peripheral veins with progressively slow blood flow and the muscles of the lower extremities in a relaxed state, resulting in slow blood flow and venous thrombosis. Besides, long-term bed rest in the process of labor and puerperium makes lower extremity activity decreasing, leading to slow speed of lower extremity blood flow, the patient's blood stasis in blood vessel wall, thus causing thrombosis. In addition, improper healing or care of lower extremity DVT can compromise postnatal recovery and result in lipid sclerosis and pigmentation in the affected limb, which may also lead to partial or complete functional loss of the affected limb, at the same time, lower extremity venous thrombosis detached to form emboli with blood flow obstructing systemic important organs, most likely causing PE, causing life-threatening, so clinically, postpartum lower extremity DVT in pregnant women with PIH should be treated and prevented aggressively. Anticoagulant therapy is a common prevention method, such as heparin, vitamin K antagonists, and so on.^[20–22]^ Besides medication, it is of great significance to cooperate with certain medical care.

The usual care model often ignores the venous flow characteristics of the lower extremities, and it is able to promote blood reflow after taking early postural intervention, effectively preventing the formation of lower extremity deep venous thrombosis in postpartum women with PIH. This is because after giving birth, the heel of the pregnant woman is raised, the lower leg is suspended, which is conducive to the lower limb venous blood reflux, thus preventing blood stasis in the lower limb veins; the calf muscle massage can promote the micro-vein dilation, improve the lower limb blood circulation, and relieve the lower limb muscle pain; by doing exercise and getting out of bed in early stage, the lower extremity blood flow can be further increased, and the purpose of preventing the lower extremity deep venous thrombosis can be achieved finally.

Clinically, it has been confirmed that postural intervention on prevention of deep venous thrombosis of lower extremity in postpartum women with PIH can help reduce the pressure on iliac veins and other iliac veins, promote blood reflux of postpartum lower limbs, and reduce the deep venous thrombosis of postpartum lower extremity in pregnant women with PIH. The clinical effect is reliable. However, the evidence from RCTs is not consistent. With the increasing number of clinical trials, it is extremely urgent to carry out systematic evaluation on postural intervention on prevention of deep venous thrombosis of lower extremity in postpartum women with PIH. In this study, we will summarize the latest evidence of postural intervention on prevention of deep venous thrombosis of lower extremity in postpartum women with PIH. This work also provides beneficial evidence for determining whether postural intervention on prevention of deep venous thrombosis of lower extremity in postpartum women with PIH is effective and safe, which is beneficial to both clinical practice and health-related decision makers.

However, this systematic review has some limitations. The included women had different modes of delivery, times of birth and gestational age, degree of hypertension and antihypertensive drugs used by the patients differed, routine care methods differed, and there may be some clinical heterogeneity. The course of the disease of the patients also varies, which may affect the results to some extent. Due to the limitation of language ability, we only search literature in English and Chinese, and may ignore research or reports in other languages.

## Author contributions

**Data collection:** Jie Chen and Lijuan Guo

**Funding support:** Yuqin Shi

**Investigation:** Shuangxi Li

**Literature retrieval:** Jie Chen and Lijuan Guo

**Software operating:** Shuangxi Li

**Supervision:** Yuqin Shi

**Writing – original draft:** Jie Chen and Lijuan Guo

**Writing – review & editing:** Jie Chen and Lijuan Guo and Yuqin Shi

## References

[1] ACOG Practice Bulletin No. 196. Thromboembolism in pregnancy. Obstet Gynecol 2018;132:e1–7. 29939938 10.1097/AOG.0000000000002706

[2] Kane EV Calderwood C Dobbie R. A population-based study of venous thrombosis in pregnancy in Scotland 1980-2005. Eur J Obstet Gynecol Reprod Biol 2013;169:223–9. 23684606 10.1016/j.ejogrb.2013.03.024

[3] Heit JA Kobbervig CE James AH. Trends in the incidence of venous thromboembolism during pregnancy or postpartum: a 30-year population-based study. Ann Intern Med 2005;143:697–706. 16287790 10.7326/0003-4819-143-10-200511150-00006

[4] Creanga AA Syverson C Seed K. Pregnancy-related mortality in the United States, 2011–2013. Obstet Gynecol 2017;130:366–73. 28697109 10.1097/AOG.0000000000002114 PMC5744583

[5] Høibraaten E Amundsen T Skjeldestad FE. Deep venous thrombosis in young women in Norway. Tidsskr Nor Laegeforen 2000;120:332–5. 10827523

[6] Tang HQ Hu SJ Zhu XS. Effect of exercise nursing combined with aspirin on blood clotting factor in patients with pregnancy induced hypertension and prevention of lower extremity deep venous thrombosis. Chin J Thromb Hemostasis 2018;24:877–8.

[7] Pană RC Pană LM Istratoaie O. Incidence of pulmonary and/or systemic thromboembolism in pregnancy. Curr Health Sci J 2016;42:283–8. 30581582 10.12865/CHSJ.42.03.08 PMC6269609

[8] Raţiu A Motoc A Păscuţ D. Compression and walking compared with bed rest in the treatment of proximal deep venous thrombosis during pregnancy. Rev Med Chir Soc Med Nat Iasi 2009;113:795–8. 20191834

[9] Wang GJ. An observation on the effect of early posture intervention on prevention of deep venous thrombosis of lower limbs by pregnant women with hypertension. Smart Healthcare 2020;6:78–80.

[10] Yu P. Effect of early postural intervention on postpartum deep venous thrombosis of lower extremities in pregnant women with hypertension. Chin J Clin Ration Drug Use 2017;10:134–5.

[11] Jia YP. An observation on the influence of early posture intervention on prevention of pregnant women with hypertension and pregnant women's postpartum deep venous thrombosis. Elec J Pract Gynecol Endocrinol 2019;6:58.

[12] Moher D Shamseer L Clarke M. Preferred reporting items for systematic review and meta-analysis protocols (PRISMA-P) 2015 statement. Syst Rev 2015;4:1. 25554246 10.1186/2046-4053-4-1 PMC4320440

[13] Gestational hypertension group, obstetrics and gynecology branch, Chinese Medical Association. Guidelines for diagnosis and treatment of hypertensive disorder complicating pregnancy (2015). Chin J Perinat Med 2016;19:161–9.

[14] James AH Jamison MG Brancazio LR. Venous thromboembolism during pregnancy and the postpartum period: incidence, risk factors and mortality. Am J Obstet Gynecol 2006;194:1311. 16647915 10.1016/j.ajog.2005.11.008

[15] Simpson EL Lawrenson RA Nightingale AL. Venous thromboembolism in pregnancy and the puerperium: incidence and additional risk factors from a London perinatal database. BJOG 2001;108:56–60. 11213005 10.1111/j.1471-0528.2001.00004.x

[16] Jacobsen AF Skjeldestad FE Sandest PM. Incidence and risk patterns of venous thromboembolism in pregnancy and puerperium- a register-based case-control study. Am J Obstet Gynecol 2008;198:233 e1–7. 10.1016/j.ajog.2007.08.041 17997389

[17] Devis P Knuttinen MG. Deep venous thrombosis in pregnancy: incidence, pathogenesis and endovascular management. Cardiovasc Diagn Ther 2017;7:S309–19. 29399535 10.21037/cdt.2017.10.08 PMC5778511

[18] Greer IA. Thrombosis in pregnancy: maternal and fetal issues. Lancet 1999;353:1258–65. 10217099 10.1016/S0140-6736(98)10265-9

[19] Sultan AA West J Tata LJ. Risk of first venous thromboembolism in and around pregnancy: a population-based cohort study. Br J Haematol 2012;156:366–73. 22145820 10.1111/j.1365-2141.2011.08956.x

[20] van Der Heijden JF Hutten BA Büller HR. Vitamin K antagonists or low-molecular-weight heparin for the long term treatment of symptomatic venous thromboembolism. Cochrane Database Syst Rev 2000;CD002001 undefined. 11034739 10.1002/14651858.CD002001

[21] Narin C Reyhanoglu H Tülek B. Comparison of different dose regimens of enoxaparin in deep venous thrombosis therapy in pregnancy. Adv Ther 2008;25:585–94. 18568442 10.1007/s12325-008-0068-0

[22] Ulander VM Stenqvist P Kaaja R. Treatment of deep venous thrombosis with low-molecular-weight heparin during pregnancy. Thromb Res 2002;106:13–7. 12165283 10.1016/s0049-3848(02)00074-9

